# Examination of Urine, Fæces, Etc.—I

**Published:** 1907-08-10

**Authors:** 


### Points in Pathology.

### EXAMINATION OF URINE, FÆCES, etc.—I.

It will not be of much use entering into the common quantitative and qualitative analyses of urine that are daily being used; they are fully described in all the text-books, and the general practitioner, we do not doubt, is well acquainted with them. When it comes, however, to the ordinary examination of the deposits in urine, and to the testing of the presence or absence of organisms there, then the standard of knowledge exhibited is not so good, and, astonishing though it may appear, many medical men are unable to diagnose casts, and mistake extraneous material for them. Again, urines are often sent to the laboratory in ordinary dirty bottles with the request that a bacteriological examination may be made on them. A few words, then, on several of these points may be found useful.

### Preliminaries.

If it is desired to examine a urine bacteriologically catheter specimens should always be obtained, this applying equally to both sexes, and the fluid should be run into some sterile receptacle. This, if not at hand, can easily be improvised as follows: Take a six-ounce or larger size medicine bottle and boil it thoroughly (cork included) for a quarter of an hour, drain off excess of water by holding bottle upside down, run in urine from catheter, cork, and send to laboratory as soon as possible. This, though not absolutely preventing contamination, reduces it at least to a minimum, and allows the expert to examine the deposit bacteriologically, or to make cultivations with some degree of certainty that he is not dealing with ordinary cocci fallen in from the air. If a sterile litre flask is at hand, or, better, one with sterile broth in it, all that is required is to quickly take out the plug, let some of the urine run in, put the plug in again, and then send at once to the laboratory. The flask with the broth can then be incubated and any growth that takes place noted, mixed infections being separated out by plating on gelatin or agar. If only an examination of the deposit is required, the boiled bottle method again is sufficient, but if a proper bacteriological examination is wanted the practitioner must remember that examining stained films is not conclusive as to what organism or organisms are present; cultures must also be made, and the individual organisms put through their different cultural tests.

### The Centrifugalising Machine.

For the study of deposits a centrifuge is essential. Many simple and cheap ones are on the market, so there is little difficulty about obtaining one. Any model that is selected should be securely fixed and carefully looked after, so as to be ready for use whenever wanted. If we are going to look at the deposit for bacteria, the tube used for centrifuging must, of course, be sterilised first, as, if not, contamination could easily take place during the process. Having finished the centrifuging, one pours off the fluid, leaving the deposit at the bottom of the tube behind, and then examines this stained or fresh. For the first method, pick up some of the deposit with a (looped platinum needle (previously sterilised by holding in the bunsen burner), smear on a slide, fix by heating over the flame, stain by any of the common stains (*vide* previous articles) or by Gram's or Ziehl-Neelsen's methods.

### Under the Microscope.

The specimen is now ready for the microscope. With the $\frac{1}{12}$-in. objective pus cells, epithelial cells, baccilli, or cocci may be easily detected; to quote only a few, tubercle bacilli, gonococci, staphylococci, the colon bacillus, and others. For the second method, some of the deposit, picked up in a similar manner, is placed on a slide and simply covered with an ordinary slip, this being ringed with vaseline or not, depending on the length of time required for the examination. By this method, one may note, if a high power is used, the different organisms in a living condition, and it is sometimes of importance to know if those are motile or not, but by far the more common use of it is to detect comparatively large bodies, such as crystals, casts, pus cells, blood cells, epithelial cells, ova of parasites, etc. For these we do not require to use such a high power, the $\frac{1}{6}$ in. objective usually being sufficient.

### Crystals in Urine.

Various different forms of crystals may be met with in the urine, and their detection and diagnosis is important in determining the condition from which the patient is suffering. Of the commoner forms of crystals one may mention those of calcium oxalate, easily told by their similarity to the backs of envelopes (the octahedral form) or to small dumb-bells, of uric acid by their brown colour and some modification of a rhombic prism as regards shape, crystalline phosphate of lime (stellar phosphates), or of triple ammonium magnesium phosphates, often described as resembling knife rests or coffin lids, in other instances like feathers. The latter are found in urines that have undergone decomposition and become ammoniacal; good examples are found in cases of spinal injuries and old-standing cystitis from other causes. Of rarer forms urate of ammonia, urate of soda, cystin, leucin, tyrosin, and cholesterin may be mentioned.

### Permanent Specimens.

For making permanent specimens it is best to use glycerin jelly as the preservative; after the cover slip has been applied remove excess of fluid with blotting paper, let stand for a day or two, then ring on a turntable with balsam or asphalt. Unless this is done properly, air will get in, or the fluid escape, especially in hot weather, so rendering the specimen of little use. The next thing an ordinary wet specimen of deposit may show are casts, and the detection of those is very important as they indicate inflammatory mischief in the kidneys.

*(To be continued.)*

